# Supplementary material for: The interactome of intact mitochondria by cross-linking mass spectrometry provides evidence for coexisting respiratory supercomplexes
Source: Mol Cell Proteomics. 2017 Dec 8;17(2):216–32. doi: 10.1074/mcp.RA117.000470 (PMC5795388; doi:10.1074/mcp.RA117.000470)
Supplement: Supplemental Data [file supp_RA117.000470_133922_0_supp_23978_fzffwf.zip › spectra_annotation/mito_spectra_annotation/255-1-2-1-3-1.pdf]

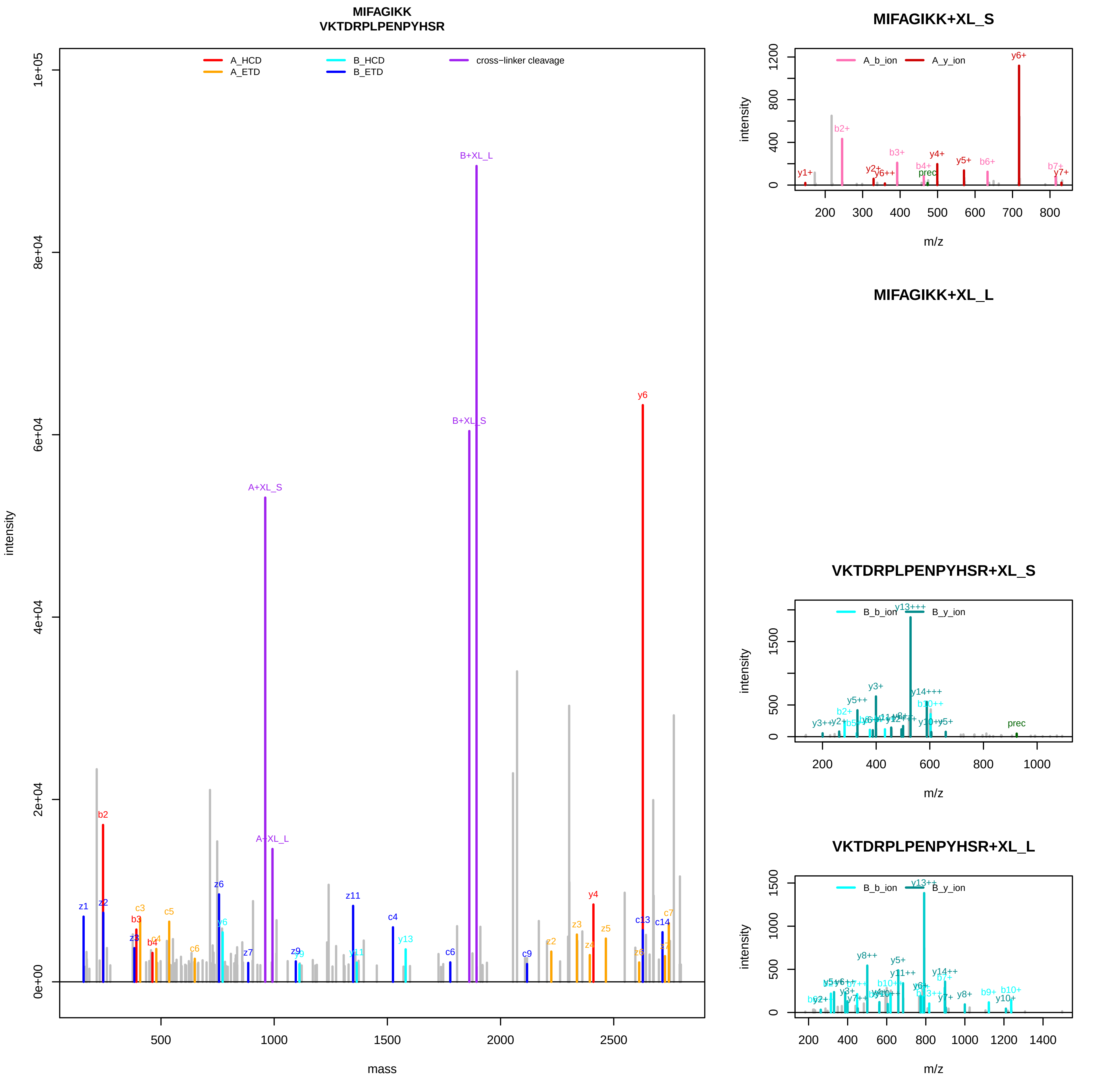
MIFAGIKK
VKTDRPLPENPYHSR
A_HCD
A_ETD
B_HCD
B_ETD
cross−linker cleavage
intensity
mass
0e+00
2e+04
4e+04
6e+04
8e+04
1e+05
500
1000
1500
2000
2500
B+XL_L
B+XL_S
A+XL_S
A+XL_L
y6
b2
z1
z2
c3
c5
b3
z3
b4
c4
c6
z6
y6
z7
z9
y9
z11
y11
c4
y13
c6
c9
z2
z3
z4
y4
z5
z6
c13
c14
c7
z7
MIFAGIKK+XL_S
A_b_ion
A_y_ion
intensity
m/z
0
400
800
1200
200
300
400
500
600
700
800
y1+
b2+
y2+
y6++
b3+
b4+
prec
y4+
y5+
b6+
y6+
b7+
y7+
MIFAGIKK+XL_L
VKTDRPLPENPYHSR+XL_S
B_b_ion
B_y_ion
intensity
m/z
0
500
1500
200
400
600
800
1000
y13+++
y3+
y5++
b2+
y3++
y2+
y14+++
b10++
y10++
y5+
prec
VKTDRPLPENPYHSR+XL_L
B_b_ion
B_y_ion
intensity
m/z
0
500
1000
1500
200
400
600
800
1000
1200
1400
y13++
y8++
y5+
y11++
y14++
b7+
y3+
y2+
y6+
y7+
y8+
b9+
b10+
y10+
